# Mechanical behavior of endocrown vs. post-and-crown: a systematic review and meta-regression analysis

**DOI:** 10.1590/0103-644020256632

**Published:** 2026-01-12

**Authors:** Sara Fraga, Gabriela de Souza Balbinot, Bruna Garcia Quadros, Stéfani Becker Rodrigues, Camila Cristina Foggi, Fabrício Mezzomo Collares

**Affiliations:** 1 Department of Conservative Dentistry, Prosthodontics Unit, School of Dentistry, Federal University of Rio Grande do Sul, Porto Alegre, RS, Brazil; 2 Dental Materials Laboratory, School of Dentistry, Federal University of Rio Grande do Sul, Porto Alegre, RS, Brazil; 3 School of Dentistry, Federal University of Rio Grande do Sul, Porto Alegre, RS, Brazil

**Keywords:** monoblock restoration, endocrown, post-and-core, crown, resistance

## Abstract

This systematic review and meta-regression analysis compared the load-to-fracture of endodontically treated teeth restored with endocrown and post-and-crown restorations, identifying factors influencing their mechanical behavior. Literature search in PubMed/MEDLINE, Web of Science, Scopus, EMBASE, and LILACS included studies published until September 2025. The search strategy combined Medical Subject Headings and free-text keywords. Two independent researchers performed the study selection. The eligibility criteria considered in vitro studies that evaluated the load-to-fracture of endodontically treated teeth restored with endocrowns and had a control group consisting of teeth restored with post-and-crown. From 862 studies, 25 met the eligibility criteria. The final meta-regression model explained 86% of the within-group variance, showing no significant differences in the load-to-fracture between endocrowns and post-and-crown restorations (p>0.05). The type of teeth, ferrule presence, and restoration material did not significantly influence the outcomes, whereas luting material and load application angle were critical determinants of load-to-fracture performance. Resin composite significantly increased the load-to-fracture compared to conventional resin cement (p<0.05). Oblique forces during mechanical tests reduced the load-to-fracture values (p<0.05). In conclusion, similar load-to-fracture performance is expected between endocrown and post-and-crown restorations. Luting material and mechanical testing parameters influenced outcomes and may have contributed to heterogeneity in prior meta-analyses.

## Resumo

A presente revisão sistemática com meta-regressão comparou a carga para fratura de dentes tratados endodonticamente restaurados com endocrown e pino-coroa, identificando fatores que influenciam seu comportamento mecânico. As buscas foram realizadas nas bases PubMed/MEDLINE, Web of Science, Scopus, EMBASE e LILACS, incluindo estudos publicados até setembro de 2025. A estratégia de busca utilizou descritores MeSH e palavras-chave livres. A seleção foi realizada por dois pesquisadores independentes. Foram incluídos estudos in vitro que avaliaram a carga para fratura de dentes tratados endodonticamente restaurados com endocrown, com grupo controle de pino e coroa. Dos 862 estudos identificados, 25 atenderam aos critérios de elegibilidade. O modelo final de meta-regressão explicou 86% da variabilidade intra-grupo, não identificando diferença significativa na carga para fratura entre endocrowns e pino-coroa (p>0,05). O tipo de dente, a presença de férula e o material restaurador não influenciaram significativamente o desfecho, enquanto o cimento e o ângulo de aplicação da carga foram determinantes. Resina composta resultou em maiores valores de carga para fratura do que cimento resinoso convencional (p<0,05). Forças oblíquas reduziram os valores de carga para fratura (p<0,05). Em conclusão, espera-se desempenho semelhante na resistência à fratura entre endocrowns e pino-coroa. O cimento e os parâmetros do teste mecânico influenciaram os resultados, podendo contribuir para a heterogeneidade reportada em metanálises anteriores.

**Figure:**
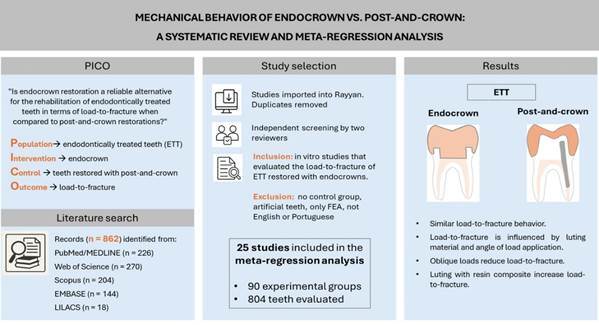

## Introduction

Endodontically treated teeth (ETT) may exhibit significant coronal destruction^1^ and a higher risk of failure compared to vital teeth^2^ ^,^ ^3^, which makes their restorative rehabilitation challenging. Traditionally, an intraradicular post combined with a full coverage crown has been used to restore both function and esthetics of severely damaged ETT^1^. However, this method may require additional dentin removal to insert the intraradicular post, which can further weaken the tooth structure^4^.

Endocrown restorations have emerged as a more conservative alternative for teeth with significant structural loss^5^. These restorations are defined as a single-unit crown that extends into the pulp chamber of an endodontically treated tooth, achieving retention through adhesive luting and specific preparation techniques^6^. Endocrown restoration requires a simpler and less invasive preparation compared to traditional post-and-core crowns, resulting in reduced treatment time and cost^1^.

Clinical evidence regarding endocrown restorations is promising. A systematic review reported an estimated 5-year survival rate of 93.8% for endocrowns in premolars and 89.1% for endocrowns in molars, with no significant difference compared to conventional crowns^7^. Primary causes of endocrown failure include loss of retention, periodontitis, and fracture of the restorative material^8^.

The biomechanical behavior of endocrown restorations has been the focus of numerous systematic reviews^8^ ^,^ ^9^ ^,^ ^10^ ^,^ ^11^. Although these reviews suggest that endocrowns exhibit comparable or even superior biomechanical performance to conventional crown restorations, they consistently highlight the considerable heterogeneity among included studies as a significant limitation, hindering the interpretation of the results. Therefore, identifying the variables that significantly impact the mechanical performance of endodontically treated teeth restored with endocrowns is crucial to understanding this high variability and guiding future research. In this context, the present study aimed to systematically review the literature on in vitro studies comparing the load-to-fracture of endodontically treated teeth restored with endocrowns versus post-and-crown restorations, identifying predictor factors of their mechanical behavior.

## Materials and methods

This systematic review was reported following the PRISMA statement^12^. The study was designed to address the following PICO question: "Is endocrown restoration a reliable alternative for the rehabilitation of endodontically treated teeth in terms of load-to-fracture when compared to post-and-crown restorations?" The population consisted of endodontically treated teeth, the intervention was endocrown restoration, the control group comprised teeth restored with post-and-crown, and the outcome of interest was the load-to-fracture.

### Search strategy

A literature search was performed in PubMed/MEDLINE, Web of Science, Scopus, EMBASE, and LILACS databases for studies published up to September 2025. The search strategy included a combination of Medical Subject Headings (MeSH) and free-text keywords (Supplementary material 1).

### Study selection

All identified studies were imported into Rayyan software^13^ for duplicate removal and subsequent selection analysis. Two authors (S.F. and B.N.Q.) independently carried out the search and selection process. Initially, studies were screened based on titles and abstracts, applying the following inclusion criteria: in vitro studies that evaluated the load-to-fracture of endodontically treated teeth restored with endocrowns. In the second stage, full-text articles were assessed, and studies were excluded if they lacked an appropriate control (teeth restored with post-and-crown), used artificial teeth, were published in languages other than English or Portuguese, or were solely based on finite element analysis. Any disagreements between reviewers were resolved by consensus.

### Data extraction

Data extraction from the eligible studies was performed by one author (S.F.) and cross-checked by a second author (G.S.B.). Extracted data included type of restoration (endocrown or post-and-crown), tooth type, presence of ferrule, finishing line, post material, material of the restoration, luting agent, presence of aging (thermocycling, mechanical cycling, or thermomechanical cycling), angle and speed of load application, the mean and standard deviation of the load-to-fracture test, besides the year of publication, and sample size.

If a study did not report the mean and standard deviation or the sample size, the corresponding authors were contacted three times via email. In the absence of a response, these studies were excluded from the meta-regression analysis.

### Quality assessment

Two authors (S.F. and B.N.Q.) independently assessed the risk of bias in the included studies using the RoBDEMAT tool^14^, evaluating four domains. In Domain 1, 'Bias in planning and allocation,' factors such as the presence of a proper control group, randomization of samples, and sample size determination were assessed. In Domain 2, 'Bias in sample/specimen preparation,' the studies were evaluated on whether they reported all necessary information for specimen preparation, including tooth preparation and the materials used. Domain 3, 'Bias in outcome assessment,' focused on describing the mechanical test characteristics, such as the material and dimensions of the piston used to apply the load, the location of load application, and the test speed. Blinding of the operator was not applicable in this context, as blinding was not feasible for the type of restoration (post-and-crown or endocrown). Domain 4, 'Bias in data treatment and outcome reporting,' examined the adequacy of the statistical analysis, including the software used, significance levels, and any selective reporting of relevant outcomes.

### Data analysis

The collected data were pooled for frequencies in each of the tested variables. The load-to-fracture data were used as the outcome variable in a random-effects inverse-variance meta-analysis to understand the effect of heterogeneity and calculate the effect sizes and standard errors in the data for each study. As the heterogeneity was too high for predictions using this model (>99%), a random-effects linear meta-regression model was applied. The type of restoration was “Endocrown” and “Post-and-Crown," and the latter was classified as the reference category. Tooth type was categorized into “Anterior” or “Posterior” (reference category). Post material was categorized to allow comparisons, being classified according to the composition, as “Glass-fiber” and “Metal” (reference category). The ferrule was considered as “Absent” (reference category), “Present”, or not reported (NR). Finish lines were classified as “Chamfer” (reference category), “Shoulder”, “Flat”, and not reported (NR). The material used for restoration was categorized according to the composition: “Feldspathic” (reference category), “Leucite”, “Lithium disilicate”, “Zirconia-reinforced lithium silicate”, “Zirconia”, or “Resin composite”. The luting agent was classified according to cement classification, namely “Conventional resin cement” (reference category), “Self-adhesive resin cement”, “Resin composite”, “Conventional resin cement for core”, or not reported (NR). If specimens were aged, they were divided into categories according to the type of aging process applied, being "Thermomechanical cycling", "Thermo cycling", or "Mechanical cycling", with values for non-aged specimens being used as the reference for the regression. The angle for load application was divided into 0º (reference category), 30º, 45º, 60º, and 90º. Load speed, sample size, and publication year were presented as continuous variables. The study ID was used as a regression moderator considering the collected variable's impact to explain the heterogeneity between studies. An initial analysis for single associations with α = 0.20 was included in the multiple meta-regression model. For this model, a significance of 0.05 was set. The model was adjusted for a Monte Carlo permutation test for meta-regression to consider possible overestimations in the model. The R^2^ was calculated to assess the proportion of between-study variance explained by the covariates, while the I^2^ was calculated to assess the residual variation due to heterogeneity. All analyses were conducted using STATA 14 (StataCorp LLC, TX, USA).

## Results

### General characteristics

**Figure 1:**
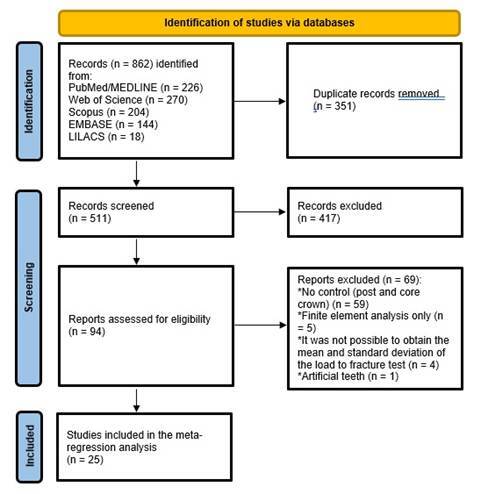
Flow diagram illustrating the study selection process according to the PRISMA statement.

Figure 1 describes the study selection process, reported according to the PRISMA statement. Database searching identified 862 records. After removing duplicates, 511 records were analyzed by title and abstract. Of these, 417 were excluded because they did not meet the inclusion criteria, and 94 were deemed eligible for full-text analysis. Following the final full-text examination, 25 studies were included in the meta-regression analysis, with 90 experimental groups and 804 teeth evaluated.

The main characteristics of the included studies are described in Supplementary Table (Boxes 1 to 7). Seventeen studies have used molars^15^ ^,^ ^16^ ^,^ ^17^ ^,^ ^18^ ^,^ ^19^, and premolar teeth^20^ ^,^ ^21^ ^,^ ^22^ ^,^ ^23^ ^,^ ^24^ ^,^ ^25^ ^,^ ^26^ ^,^ ^27^ ^,^ ^28^ ^,^ ^29^ ^,^ ^30^ ^,^ ^31^, while eight studies have been conducted on anterior teeth^32^ ^,^ ^33^ ^,^ ^34^ ^,^ ^35^ ^,^ ^36^ ^,3,^ ^7^ ^,^ ^38^ ^,^ ^39^.

Different conditions of ferrule were considered, including no ferrule and ferrules ranging between 0.5 and 2 mm in height. Regarding endocrown preparation, the pulp chamber depth ranged between 2 and 10 mm, but most of the studies have used a depth of 4 mm^15^ ^,^ ^16^ ^,^ ^20^ ^,^ ^23^ ^,^ ^35^ and 5 mm^25^ ^,^ ^27^ ^,^ ^28^ ^,^ ^29^ ^,^ ^36^. The most frequently used finishing lines were chamfer, shoulder, and flat surface (butt margin).

The restorative materials evaluated included machined resin composite and ceramics, with the lithium disilicate-reinforced glass ceramic being the most used material ^15^ ^,^ ^16^ ^,^ ^17^ ^,^ ^18^ ^,^ ^20^ ^,^ ^21^ ^,^ ^23^ ^,^ ^24^ ^,^ ^25^ ^,^ ^30^ ^,^ ^33^ ^,^ ^34^ ^,^ ^35^ ^,^ ^36^ ^,^ ^37^ ^,^ ^38^ ^,^ ^39^. One study also investigated metal restorations^31^. The indirect restorations were luted using conventional resin cement in 16 studies^15^ ^,^ ^18^ ^,^ ^19^ ^,^ ^20^ ^,^ ^21^ ^,^ ^22^ ^,^ ^23^ ^,^ ^25^ ^,^ ^27^ ^,^ ^28^ ^,^ ^29^ ^,^ ^31^ ^,^ ^33^ ^,^ ^35^ ^,^ ^37^ ^,^ ^38^, self-adhesive resin cement in 4 studies^17^ ^,^ ^24^ ^,^ ^36^ ^,^ ^39^, resin composite in 4 studies^16^ ^,^ ^26^ ^,^ ^30^ ^,^ ^34^, and conventional resin cement for core in one study^32^.

Some studies have performed aging before the load-to-fracture test, including thermomechanical cycling^15^ ^,^ ^16^ ^,^ ^19^ ^,^ ^20^ ^,^ ^21^ ^,^ ^23^ ^,^ ^29^ ^,^ ^30^ ^,^ ^35^, thermocycling^17^ ^,^ ^25^ ^,^ ^33^ ^,^ ^34^ ^,^ ^37^, and just mechanical cycling^28^ ^,^ ^39^. The aging protocols differed significantly regarding the number of thermal and mechanical cycles employed. Most studies used a metal ball with varying diameters to apply the load at speeds of 0.5 mm/min and 1 mm/min for the load-to-fracture test.

### Quality assessment

**Figure 2:**
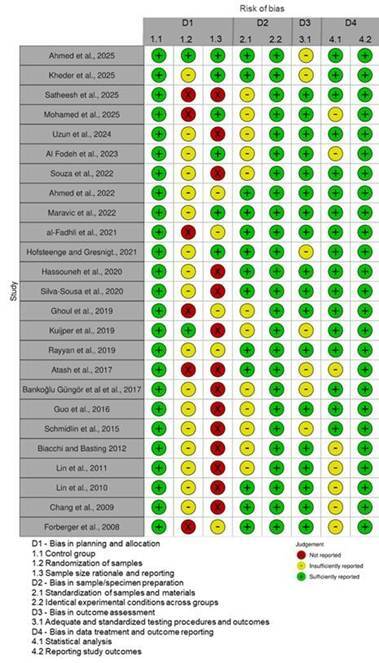
Quality assessment performed according to the RoBDEMAT tool.

Figure 2 presents the quality assessment results conducted using the RoBDEMAT tool. In Domain 1, 'Bias in planning and allocation,' the primary issues identified were the lack of description of how randomization was performed and the absence of a sample size calculation. In Domain 2, 'Bias in sample/specimen,' some studies did not provide adequate details on sample preparation, such as the type of finishing line and a complete description of the ferrule characteristics (height and thickness). Besides, some studies did not report the commercial brand of the materials employed. In Domain 3, 'Bias in outcome assessment,' some studies failed to report key aspects, such as the material and dimensions of the piston used for load application. In Domain 4, 'Bias in data treatment and outcome,' the issue was the omission of the software used for statistical analysis.

### Meta-regression analysis

The final meta-regression model included 90 groups, extracted from the 25 included studies, and explained 86% of the between-study variance (R² = 86%). The within-group variance was statistically significant (p < 0.01). Residuals were homoscedastic and normally distributed (p > 0.05), and no outliers that exert leverage in the data were found. The variables were selected based on their potential to serve as moderator variables. All selected variables are listed in Supplementary Material 2, where univariate analyses are presented. From these results, some moderators were excluded from the final meta-regression model as they did not show influence in the univariate screening (post material and finish line; p > 0.2). The type of restoration was retained in the final model because it was the primary focus of interest, along with the restoration material.

The results of the final meta-regression model are presented in Table 1. The analysis showed that the type of restoration (post-and-crown vs. endocrown) did not significantly affect the load-to-fracture of endodontically treated teeth (p > 0.05). Similarly, the presence of a ferrule did not significantly influence the load-to-fracture (p > 0.05). The model identified that the luting agent significantly influenced the load-to-fracture of teeth restored with either endocrown or post-and-crown. Using resin composite significantly increased the load-to-fracture compared to conventional resin cement.

In terms of aging, none of the protocols (thermomechanical cycling, thermocycling alone, or mechanical cycling) affected load-to-fracture. Regarding the mechanical test, the incidence of the load application significantly influenced the results. Oblique forces and 1mm/min load speed were associated with reduced load-to-fracture values.

## Discussion

Endocrown popularity is mainly due to its less invasive approach for restoring ETT. Clinical and laboratory analyses compared endocrown to post-and-crown restorations. However, the meta-analytic evaluation of these studies' pooled data showed high heterogeneity and limited strength to support a reliable evidence-based decision-making process for using either restorative strategy ^11^ ^,^ ^40^. To understand the variables associated with the load-to-fracture in restored ETT and address the heterogeneity of meta-analytic data, the present study employed a meta-regression analysis of 25 studies, with 90 groups and 804 analyzed teeth. Our final model included variables that explained 86% of the variance among the included studies. The main finding is that the load-to-fracture of ETT is not predicted by the restoration with either endocrown or post-and-crown (Coef: -3.98 N. CI 95% -150.79; 142.82). The luting material and the load application angle were variables that significantly modified the load-to-fracture in the analyzed data.

The meta-regression analysis showed that endocrown restorations have a similar load-to-fracture behavior to the traditional post-and-crown approach for severely damaged teeth (Table 1). These findings agree with other reports in the literature^8^ ^,^ ^9^ ^,^ ^10^ ^,^ ^11^. The meta-analysis performed by Sedrez-Porto et al.^11^, comparing the load-to-fracture of teeth restored with endocrowns to teeth restored with conventional treatments (intraradicular posts, direct composite resins, inlay/onlay), favored the endocrown restorations, which showed higher levels of load-to-fracture. However, in a subgroup analysis, when the load-to-fracture of posterior teeth restored with endocrowns was compared to that of teeth restored with post-and-crown, there was no difference between these restorative approaches. Therefore, restoring teeth with an endocrown or post-and-crown predicts similar load-to-fracture.

The type of teeth (anterior or posterior) did not significantly influence the load-to-fracture of severely damaged ETT restored with endocrown or post-and-crown. Originally, endocrowns were developed to rehabilitate posterior teeth with a significant amount of coronal destruction and limited interocclusal space^5^. However, this type of restorative alternative in anterior teeth is questionable. A recent systematic review and network meta-analysis of in vitro studies showed that endocrowns in anterior teeth had a similar or superior mechanical performance to conventional restorative options, with a lower risk of irreparable failures^9^.

**Table 1:** Meta-regression analysis of the load-to-fracture results of endodontically treated teeth restored with endocrown and post-and-crown restorations.

Variable	Categories	Regression coefficient (in N)	Standard Error	95% CI		p-value
Type of restoration	Post-and-crown (reference)					
	Endocrown	-3.98	73.46	-150.79	142.82	1
Type of tooth	Posterior (reference)					
	Anterior	-140.12	177.65	-495.13	214.88	1
Ferrule	No ferrule (reference)					
	Yes	-111.34	86.79	-284.78	62.10	1
	NR	-124.57	351.38	-826.75	577.61	1
Restoration material	Feldspathic (reference)					
	Leucite	1288.67	686.33	-82.86	2660.19	0.535
	Lithium disilicate	-1119.06	410.32	-1939.02	-299.09	0.274
	Zirconia-reinforced lithium silicate	-121.22	123.13	-367.29	124.84	1
	Zirconia	66.50	132.09	-197.45	330.46	1
	Resin composite	-41.72	111.87	-265.27	181.83	1
	Metal	-111.90	248.29	-608.08	384.27	1
Luting agent	Conventional resin cement (reference)					
	Self-adhesive resin cement	439.56	193.55	52.77	826.35	0.391
	Resin composite	736.49	312.41	112.19	1360.79	0.036
	Conventional resin cement for core	500.06	372.20	-243.72	1243.84	0.731
Aging	No aging (reference)					
	Thermomechanical cycling	-220.56	175.33	-570.92	129.81	0.919
	Thermo cycling	-257.70	139.38	-536.23	20.83	0.948
	Mechanical cycling	91.08	202.23	-313.05	495.21	0.990
Load angle	0º (reference)					
	30º	-992.88	202.26	-1397.06	-588.70	0.011
	45º	-1501.97	206.72	-1915.07	-1088.87	0.001
	60º	-2994.27	785.43	-4563.83	-1424.72	0.024
	90º	-1129.31	193.34	-1515.68	-742.94	0.001
Load speed (mm/min)	0.5 (reference)					
	1	-937.42	321.22	-1579.33	-295.51	0.047
	3	-3563.27	1203.5	-5968.27	-1158.27	0.078
	30	-741.50	358.71	-1458.33	-24.67	0.296
	NR	33.58	198.90	-363.90	431.05	1
Year	-	-89.13	33.58	-156.24	-22.02	0.102
Sample size	-	-41.56	29.31	-100.12	17.00	0.953
I^2^ residual						84%
R^2^						86%

In the present study, a ferrule ranging from 0.5 to 2 mm height did not significantly influence the load-to-fracture behavior of severely damaged ETT restored with either endocrowns or post-and-crown systems. These findings contrast with a meta-analysis indicating that post-restored teeth with a ferrule exhibit greater fracture resistance than those without a ferrule^41^. However, that analysis reported high heterogeneity (I²=92%), suggesting that factors beyond the presence or absence of a ferrule - such as post types, crown materials, and variations in ferrule design and height - may explain the observed differences among groups. This interpretation is supported by systematic reviews of clinical trials, which indicate that the impact of the ferrule on the clinical performance of post-and-crown restorations remains controversial^42^ ^,^ ^43^ ^,^ ^44^. A clinical study with a 17-year follow-up found that substantial dentin height-defined as greater than 75% of the circumferential dentin wall with a minimum 1 mm thickness and at least 1 mm above the gingival level-was associated with higher survival rates, but only in teeth restored with prefabricated metal posts and composite cores with a covering metal-ceramic crown^45^. Furthermore, Skupien et al.^41^ observed that while ferrules improved clinical survival in premolars, no statistically significant differences in survival were found in molars and anterior teeth with or without a ferrule.

The preparation of an endocrown restoration usually involves a circumferential butt-joint margin, with no ferrule^46^ ^,^ ^47^. The effect of ferrule on the mechanical behavior of endocrown restoration remains contradictory. A recent study showed that zirconia-reinforced lithium silicate endocrowns without a ferrule have higher fracture strength than those with a 1 mm ferrule design^48^. Bamajboor and Dudley^49^ reported that the presence of a ferrule had no significant effect on the load-to-fracture of monolithic zirconia endocrowns. On the other hand, the presence of a ferrule was associated with higher fatigue failure loads in lithium disilicate endocrowns^35^. These findings suggest that further research is needed to clarify the impact of endocrown preparation design on the mechanical behavior of severely damaged ETT.

Furthermore, the authors suggest that future studies provide a more detailed description of ferrule characteristics, including not only the height but also the thickness of the remaining dentin. This would contribute to a better understanding of this factor's influence on the mechanical behavior of restored teeth.

In the meta-regression analysis, the restorative material did not significantly influence the load-to-fracture of ETT restored with endocrowns or post-and-crown restorations. This finding contrasts with the existing literature, which generally reports superior mechanical performance for reinforced glass ceramics and polycrystalline ceramics compared with feldspathic ceramics^50^. This result may be related to the influence of a single study, with a high sample size (n=20) that evaluated feldspathic ceramics and reported high fracture resistance values^19^. In addition, factors such as material thickness or variations in the mechanical testing parameters among studies, especially load application angle, may have overridden the effect of the restorative material itself.

Regarding clinical performance, a meta-analysis indicated that single crowns made of feldspathic ceramics have a significantly lower 5-year survival probability than metal-ceramic crowns. However, crowns made of reinforced glass ceramics, as well as polycrystalline ceramics, showed survival rates similar to those of metal-ceramic crowns^51^. For endocrown restorations, a clinical study found no difference in the survival rates of endocrowns made from lithium disilicate, zirconia, and polymer-infiltrated hybrid ceramic over a 2-year follow-up period^52^. The same occurred for endocrowns made of felspathic ceramic, zirconia-reinforced lithium silicate, and lithium disilicate, which presented a similar clinical behavior after 2 years^53^.

ETTs restored with either endocrowns or post-and-crown restorations luted with resin composite showed higher load-to-fracture values than those cemented with conventional resin cement. Resin composites have been proposed for luting indirect restorations due to their enhanced mechanical properties, a wide range of shades, and lower cost compared to resin cements^54^. The higher load-to-fracture values observed for the resin composite may be attributed to its greater filler content, which could reduce stress concentration and attenuate crack propagation within the restorative material during mechanical loading^55^. However, factors such as film thickness, viscosity, degradation resistance, and user-friendly characteristics should also be considered beyond load-to-fracture performance when selecting the optimal luting material.

Mechanical cycling, thermocycling, and thermomechanical cycling did not significantly affect the load-to-fracture of ETT restored with endocrown or post-and-crown systems. The aging protocols varied considerably among the studies. The number of thermocycles ranged from 1,500 to 10,000, with temperatures between 5 ºC and 55 ºC. For studies incorporating thermocycling and load cycling, the number of load cycles varied from 20,000 to 1,200,000, with most studies applying a load of 50 N. There is currently no consensus regarding the most appropriate aging protocol to replicate the oral environment accurately. According to Wiskott et al.^56^, one million loading cycles may simulate approximately one year of functional performance, while 10,000 thermal cycles may correspond to one year in function^57^. One could think that increasing the number of thermal and/or mechanical cycles, a decreased load-to-fracture will occur. However, when an adjusted multivariate analysis was performed in this study, other factors probably overcame the aging effects; further, the high non-standardization of in vitro protocols led to a lack of differentiation among studies.

The present meta-regression analysis highlighted the influence of the mechanical test parameters on the load-to-fracture of ETT. The angle of load application had a significant effect on the load-to-fracture of ETT, with oblique loads resulting in lower strength values. These findings are supported by finite element analyses, which demonstrate higher stress concentrations in teeth subjected to oblique loads^58^ ^,^ ^59^. Thus, oblique loading can be considered a challenging scenario for ETT restored with either endocrowns or post-crown.

Most studies have employed crosshead speeds of 0.5 to 1 mm/min, while a few have used speeds of 3 mm/min^27^ ^,^ ^28^ and 30 mm/min^17^ ^,^ ^29^. Meta-regression analysis indicated that test speed significantly influences the load-to-fracture. These findings agree with the study of Naves et al.^60^, who observed that higher crosshead speeds are predictive of decreased fracture resistance in premolars. This behavior may be due to the reduced ability of the structure to dissipate stress at elevated speeds.

Regarding the risk of bias assessment, most studies failed to report how randomization was performed and the sample size rationale, which is essential to ensure the validity of the results. Regarding the sample preparation, some information was missed in some studies, such as the finishing line, ferrule height, pulp chamber depth in endocrown preparation, and cement used. Some variables related to mechanical testing were also missing, such as the load application site, the material, and the size of the piston. The absence of this information reduces the study's reproducibility and may limit the generalization of the results. The limitation in the provided information resulted in the presence of a "Not reported" group in our analysis, and, despite the impact on the discussion of the evidence, it may have interfered with the adjustment of the final meta-regression model. The unexplained portion of the results may be related to this data and unknown bias in the analysis.

The heterogeneity is a standard limitation in predictions made by meta-analyses^61^, and the number of variables involved in the treatment of ETT could explain the high heterogeneity found when analyzing the load-to-fracture in this study (>99%). The final meta-regression model resulted in an R² of 86%, which represents a good fit for regression curves with 14% of the load-to-fracture behavior of ETT restored with endocrowns and post-and-crowns remaining unexplained. To ensure little impact of overestimations from the meta-regression model, a Monte Carlo permutation was performed. The *p*-values in Table 1 are already adjusted to account for the possible Type I error on the significance of moderators. Residual heterogeneity in the final model was 83.93%, which shows that between-study variation is still present in the analysis. This residual heterogeneity may be related to other testing variables that were not collected, lack of reporting in the study, as observed in the “not reported” data (Supplementary Table - Boxes 1, 2, 3, 4, 5, 6 to 7), and the biases observed in the risk of bias assessment (Figure 2). This highlights the need for standardization in testing and reporting data, allowing comparisons between approaches in the literature. While this residual heterogeneity is observed, the reduction is an important improvement, as a high R^2^ is observed, and >99% of heterogeneity was obtained in the meta-analysis, when no moderators were considered in the model (i.e., prior meta-regression analysis).

Despite the limitations of the data and model, the final model was suitable for addressing the variables that may be interfering with the load-to-fracture in restored ETT, showing that moderators are influencing the outcomes when testing endocrown vs post-and-crowns. The analysis is helpful in the design of new studies that should focus on understanding the influence of other variables, such as the characteristics of endocrown preparation and surface treatments, on the mechanical behavior of restored teeth. Also, future analysis on clinical parameters may be performed with similar models.

From a clinical perspective, the present study indicates that endocrowns and post-and-crown restorations provide comparable load-to-fracture performance for the rehabilitation of ETT. The most significant reduction in the load-to-fracture under oblique loading emphasizes the need for careful occlusal adjustment, particularly during eccentric movements, to minimize undesired lateral forces on these teeth. Additionally, special attention should be given to the luting procedure, which plays a relevant role in the mechanical behavior of both restoration types.

## Conclusion

The main findings of the present study indicate that endocrown restorations exhibit a load-to-fracture behavior similar to post-and-crown restorations in the rehabilitation of endodontically treated teeth (ETT). Factors such as luting agent and load application angle influence the load-to-fracture outcomes of ETT restored with either endocrowns or post-and-crowns systems. Proper selection of cementation technique appears essential to ensure optimal mechanical performance in these restorative approaches.

## Figures and Tables

**Box 1:**
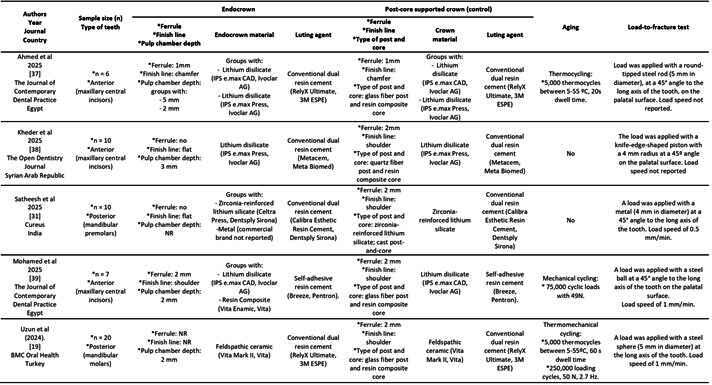
Characteristics of the included studies.

**Box 2:**
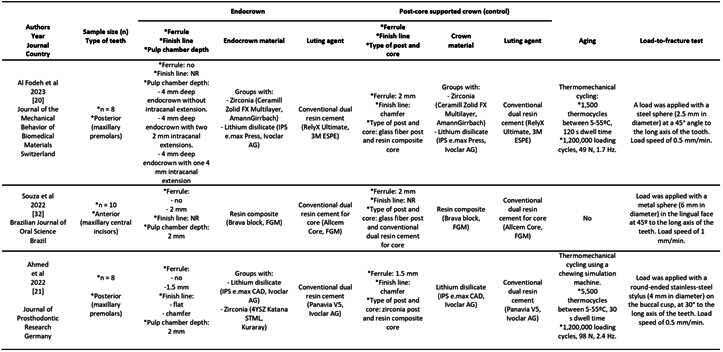
Characteristics of the included studies.

**Box 3:**
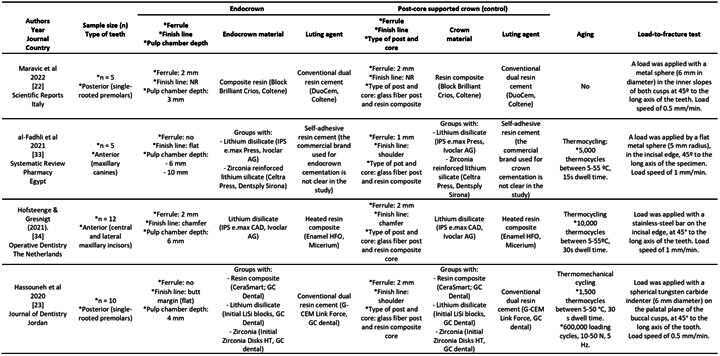
Characteristics of the included studies.

**Box 4:**
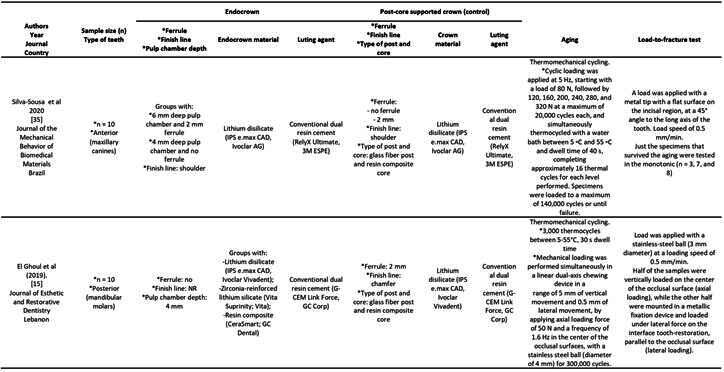
Characteristics of the included studies.

**Box 5:**
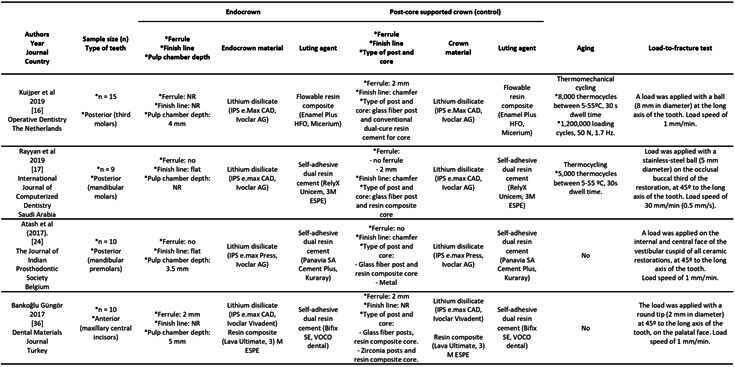
Characteristics of the included studies.

**Box 6:**
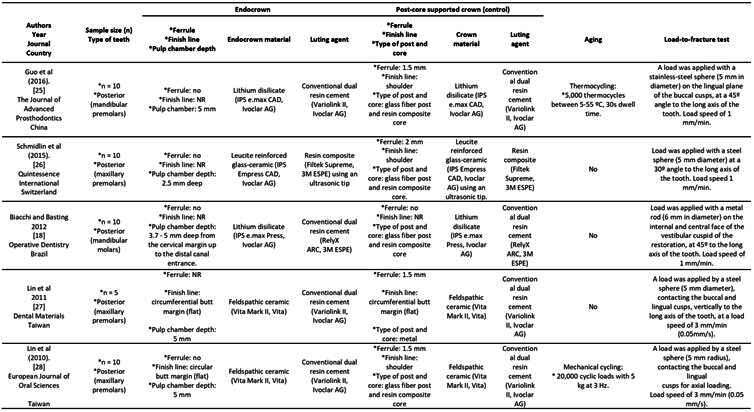
Characteristics of the included studies.

**Box 7:**
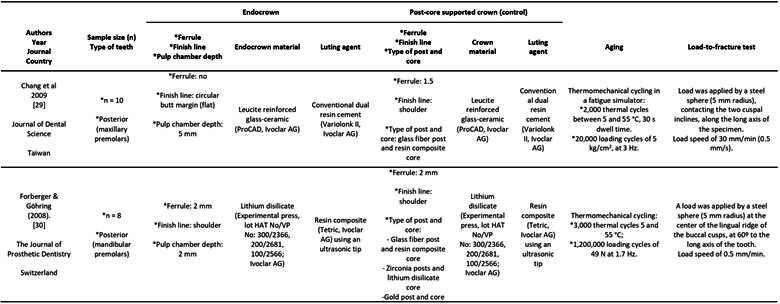
Characteristics of the included studies.
